# Antidepressive-Like Property of Dichloromethane Fraction of *Pimenta pseudocaryophyllus* and Relevance of Monoamine Metabolic Enzymes

**DOI:** 10.1155/2013/659391

**Published:** 2013-01-09

**Authors:** James Oluwagbamigbe Fajemiroye, José Luís Rodrigues Martins, Paulo César Ghedini, Pablinny Morreira Galdino, Joelma Abadia Marciano de Paula, José Realino de Paula, Fabio Fagundes Da Rocha, Elson Alves Costa

**Affiliations:** ^1^Department of Physiological Sciences, Federal University of Goiás, Campus Samambaia, 74001-970 Goiâania, GO, Brazil; ^2^Laboratory of Neuropharmacology, Department of Pharmacology, CEP 88040-970 BLOC D CCB, Federal University of Santa Catarina, UFSC, Florianópolis, SC, Brazil; ^3^Sciences and Technology Unit, Goias State University, BR 153, No. 3105, CEP 75132-903, Fazenda Barreiro do Meio, Anápolis, GO, Brazil; ^4^Faculty of Pharmacy, Federal University of Goiás, Setor Universitário, CEP 74001-970 Goiâania, GO, Brazil; ^5^Department of Physiological Sciences, Institute of Biology, Federal Rural University of Rio de Janeiro, BR465, km 07, Seropédica, 23890-000 Rio de Janeiro, RJ, Brazil

## Abstract

*Pimenta pseudocaryophyllus* popularly referred to as craveiro is considered as a calming agent in different local preparations. The present study attempted to examine antidepressant-like effect of dichloromethane fraction (DF) and role of monoamine oxidase (MAO), tryptophan, and tyrosine hydroxylase. Based on the research focus, tail suspension (TS), forced swimming (FS), and open field (OF) tests were conducted after oral administration of DF (125, 250, or 500 mg/Kg). Ex vivo assay of MAO was also conducted to evaluate inhibitory effect of DF (250 mg/Kg). Administration of DF elicits antidepressant-like response in the TS and FS. However, DF 500 mg/Kg did not alter mice performance in these models. The data obtained in the OF showed a reduction in total crossing and rearing activity; these effects suggest motor interference in TS and FS performance. Mice pretreatment with p-chlorophenylalanine methyl ester (PCPA) (100 mg/kg, i.p.—serotonin biosynthesis inhibitor) for 4 consecutive days or acute administration of **α**-methyl-p-tyrosine (AMPT) (100 mg/kg, i.p.—catecholamine synthesis inhibitor) blocked anti-immobility effect of DF in the FS. In ex vivo assay of MAO, DF did not inhibit catabolic activity of MAO. Our findings support antidepressant-like activity of DF and suggest an effect that depends on monoamine biosynthesis.

## 1. Introduction

*“Pimenta pseudocaryophyllus”* (Gomes) L.R. of Myrtaceae family is popularly known as “craveiro-do-mato,” “craveiro,” “louro-cravo,” “cataia,” “chá-de-bugre,” and “louro” and “pau-cravo” [1, 2]. The genus *Pimenta *consists of approximately 15 known species of which only *Pimenta pseudocaryophyllus* occurs in Brazilian flora. Popular applications (tranquilizer, nerve tonic, cold relief, diuretic, aphrodisiac, digestive, and menstrual realized. The FS was realized following the treatment procedure unproblems) of its leaf extract in different preparations have been reported especially in the county of Campos do Jordão, São Paulo, Brazil [1–6].

Previous neuropharmacological screening of essential oil, ethanolic leaf extract, and active fractions of the *Pimenta pseudocaryophyllus* leaf demonstrated behavioural alterations in the open field, elevated plus maze, light dark box, and barbiturate sleep induction tests without any form of motor incoordination [7–9]. Evaluation of the antidepressive-like property of dichloromethane fraction (DF) of *Pimenta pseudocaryophyllus *is borne out of its anxiolytic property that has been associated with monoaminergic receptor [8]. 

The hypothesis of biogenic amine involvement in depression has produced several generations of antidepressant agents (monoamine oxidase inhibitors (MAOIs), tricyclic antidepressants (TCAs), selective serotonin reuptake inhibitors (SSRIs), selective noradrenaline reuptake inhibitor (SNRI), and atypical antidepressants among others). The fact that clinical responses to drug effects take weeks of sustained treatment [10] and occurrence of plethora of side effects make discovery of new compounds inevitable. Meanwhile, considering the fact that conventional antidepressant and natural products have demonstrated efficacy in the clinical treatment and experimental model of anxiety [11–20], anxiolytic-like property of dichloromethane fraction obtained from ethanolic leaf extract of *Pimenta pseudocaryophyllus* makes evaluation of its antidepressant effect imperative. 

Thus, we hypothesize that the efficacy of aforementioned antidepressive drugs or natural products to treat anxiety or vice versa is a function of the active principles in these compounds and underlining mechanism of their neuropharmacological action. In this manner, the aim of this study was to investigate antidepressant-like property of DF on mice and involvement of metabolic enzymes (tryptophan hydroxylase (TrOH), tyrosine hydroxylase (TOH), and monoamine oxidase (MAO)). 

## 2. Material and Methods

### 2.1. Preparation of DF and Phytoconstituent Analysis by HPLC

The leaf collection, identification, voucher specimen (herbarium) deposit, extraction, and partitioning of ethanolic leaf extract to obtain DF as well as qualitative and quantitative analysis of this fraction by HPLC were achieved following the procedure described in our previous work [8].

### 2.2. Animals

Male albino Swiss mice (30 ± 5 g) were provided by Central Animal House of Federal University of Goiás (UFG). They were housed under controlled environmental conditions (22 ± 3°C, 12 h light/dark cycle) and allowed free access to standard food and water. All experimental procedures were conducted with strict adherence to the regulations of ethical principles in animal research as adopted by the Brazilian society of laboratory animal science. The experimental protocol was approved by research ethic council of the Federal University of Goiás (no. 104/08). 

### 2.3. Drugs and Administration

p-Chlorophenylalanine methyl ester (PCPA, 100 mg/kg) and *α*-methyl-p-tyrosine (AMPT, 100 mg/kg) used to deplete monoamine (indoleamine and catecholamine, resp.) were administered intraperitoneally to groups of mice; imipramine (IMI, 30 mg/kg), diazepam (DZP, 5 mg/kg, Cristália, Brazil), clorgyline 15 mg/kg (an irreversible and selective inhibitor of monoamine oxidase A), and tranylcypromine 15 mg/kg (a nonselective and irreversible inhibitor of monoamine oxidase) used as standard drug were dissolved in 0.9% saline, dichloromethane fraction (DF, 125 to 500 mg/kg) was dissolved in vehicle (2% polyoxyethylene sorbitan monooleate in 0.9% saline) while control animals received appropriate equivalent vehicle. Oleanolic acid (Sigma, EUA) was used as a standard for HPLC analysis of DF. All solutions were freshly prepared on test days and administered (10 mL/kg of mice body weight).

### 2.4. Pharmacological Procedure

#### 2.4.1. Tail Suspension Test (TS)

According to the method described by Steru and his collaborators [21], duration of immobility following DF treatment was measured over 6 min of TS. In this model, mice were suspended 50 cm above the floor by adhesive tape placed approximately 1 cm from the tip of the tail. Mice were treated orally with DF (125, 250, or 500 mg/kg), IMI 30 mg/kg, or vehicle prior to TS.

#### 2.4.2. Forced Swimming Test (FS)

Assessment of antidepressant-like effect of DF was conducted in FS. Pretrial exposure (15 min, 24 h prior to the test) of mice to this apparatus was followed by a 6 min test period during which scoring of immobility time was realized. The FS was realized following the treatment procedure under Section  2.4.1 and placement of mice in a cylinder (42 cm high, 18 cm in diameter) filled with water (24°C) up to 30 cm. Experimental subject was considered to be immobile when it ceased struggling and making minimum movements necessary to keep afloat [22]. In subsequent experiment mice were pretreated intraperitoneally with, 0.9% saline, PCPA 100 mg/kg for four consecutive days, or AMPT 100 mg/kg 4 hours prior to the oral administration of vehicle, DF (250 mg/kg), or IMI 30 mg/kg and were subjected to forced-swimming test to examine the effects of indoleamine or catecholamine depletion, respectively.

#### 2.4.3. Open-Field Test (OF)

Mice were individually placed at the centre of the open field apparatus in a sound proof experimental room to measure rearing and locomotor activity during 5 min after oral administration of vehicle, DF (500 mg/kg), or DZP (5 mg/kg). The floor of the OF is divided into 8 sectors of equal area. The number of crossing and rearing was registered for further statistical analysis. The apparatus was cleaned with 10% alcohol after mouse exposure.

#### 2.4.4. Ex Vivo MAO Assay by Spectrophotometric Method

Mice were treated acutely with DF (250 mg/kg), clorgyline 15 mg/kg (a selective inhibitor of MAO-A), or tranylcypromine 15 mg/kg (a nonselective and irreversible inhibitor of monoamine oxidase) and sacrificed by decapitation after 60 min. Brain tissues homogenates were prepared according to [23] and stored under −20°C in aliquots and used as the source of MAO within 48 h. Enzymatic activity was measured according to [24]. Protein concentration was estimated by using Bradford method [25]. 

### 2.5. Statistical Analysis

In order to compare level of significance between two groups, unpaired Student's *t*-test was used as described by Drummond and Tom [26, 27]. To compare more than two groups, we used ANOVA followed by Dunnett's test to compare test with control group or Student-Newman-Keuls to compare all pairs of means. All values of *P* < 0.05 were considered to be significant.

## 3. Results

**Figure 1:**
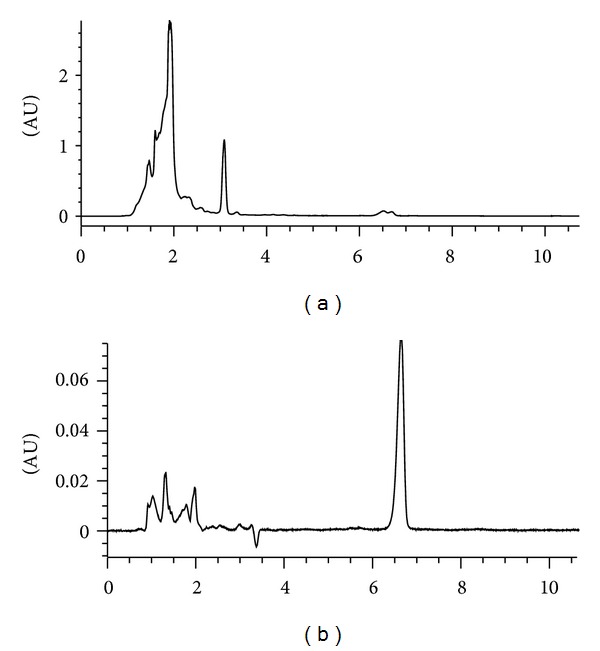
HPLC chromatogram showing (a) oleanolic acid peak detected in dichloromethane fraction of *Pimenta pseudocaryophyllus *and (b) reference drug (oleanolic acid, Sigma).

**Figure 2:**
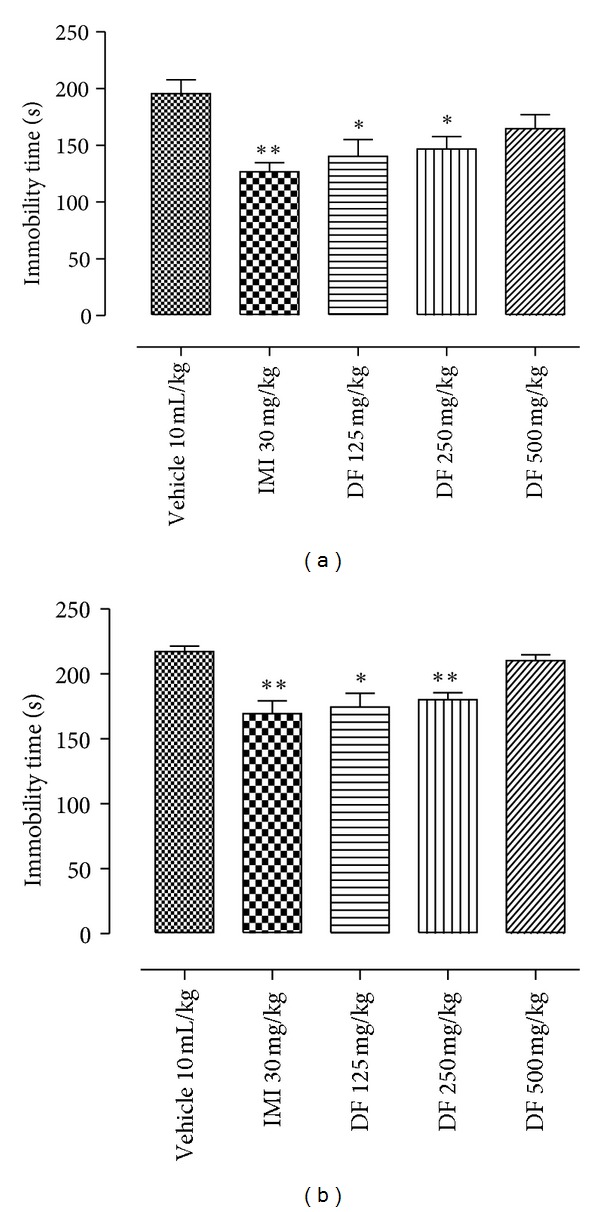
The effect of dichloromethane fraction (DF), imipramine (IMI), or vehicle administration on the immobility (a) in the TS; (b) FS. Data are presented as mean of immobility time in seconds ± S.E.M. (*n* = 10). All differences from the control group are considered to be significant at *P* < 0.05, or *P* < 0.01 as denoted by ∗ or ∗∗, respectively. Except for control group, lack of symbol ∗ on the bar indicates *P* > 0.05.

**Figure 3:**
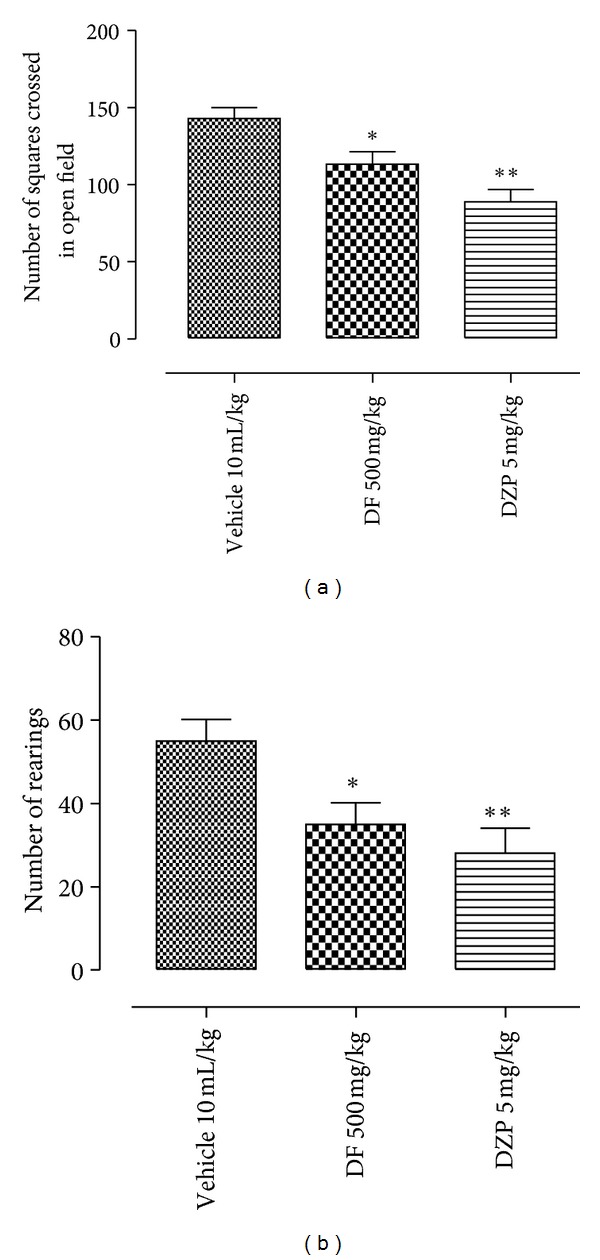
Effect of dichloromethane fraction (DF), diazepam (DZP), or vehicle oral treatments in the open-field test. Values are expressed as mean ± S.E.M (*n* = 10 ± 2).

**Figure 4:**
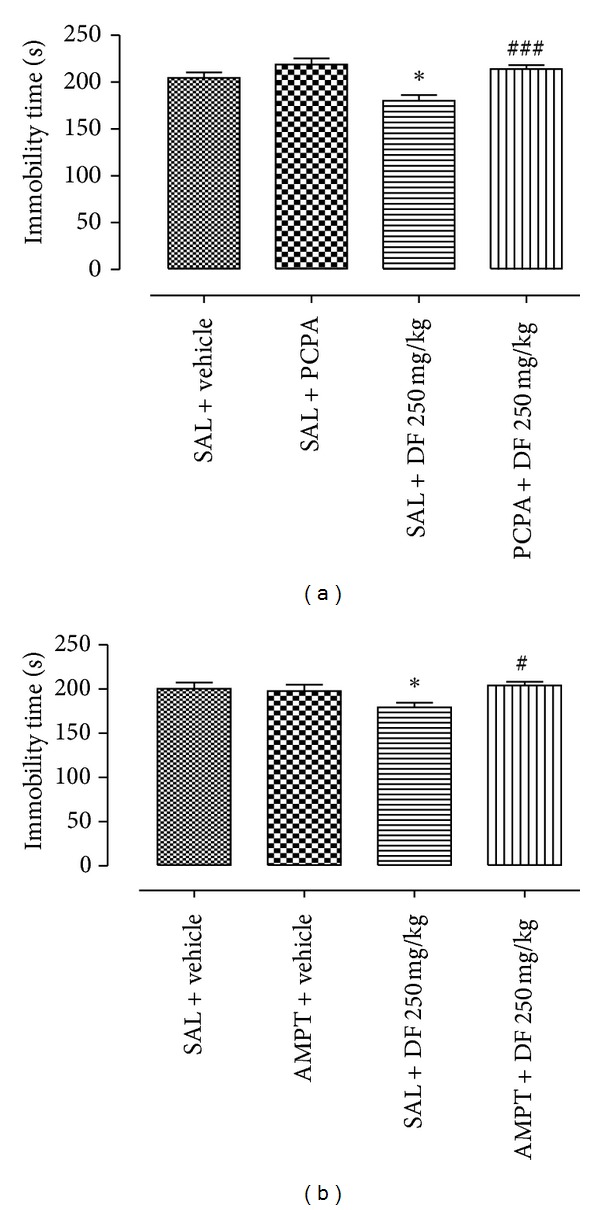
The effect of dichloromethane fraction (DF), imipramine (IMI), or vehicle administration on the immobility after pretreatment with (a) PCPA 100 mg/kg; (b) AMPT 100 mg/kg in FS. Data are presented as mean of immobility time in seconds ± S.E.M. (*n* = 10 ± 2). All differences from the control group are considered to be significant at *P* < 0.05 or *P* < 0.01 as denoted by ∗ or ∗∗, respectively, while # (*P* < 0.05) or ### (0.001) represents reversion of anti-immobility effect by PCPA or AMPT pretreatments. Except for control group, lack of symbol ∗ or # on the bar indicates *P* > 0.05.

The chromatograms in Figure 1(a) (sample DF) and Figure 1(b) (reference drug, oleanolic acid) showed relative composition of oleanolic acid (OA) to be 7.82% with respect to the concentration of DF injected (relative composition of OA = [(Cr × As × 100) ÷ (Cs × Ar)%] where Cr is concentration of reference drug, Ar is area under reference drug curve, Cs is concentration of sample, As is area under sample curve). In the pharmacological tests; like imipramine (30 mg/kg), DF (125 or 250 mg/kg) significantly reduced immobility in the TS and FS as shown in Figures 2(a) and 2(b), respectively. As shown in Figures 3(a) and 3(b), DF 500 mg/kg and diazepam (DZP 5 mg/kg) altered the number of crossings (*P < 0.05 and _ _**P < 0.01, resp.) and rearings (_ _*P < 0.05 and _ _**P < 0.01, resp.) in the open-field test (OF) significantly when compared with the vehicle treated group. Reduction in these parameters is an indication of motor incoordination by DF 500 mg/kg or DZP 5 mg/kg treatment. Effect of biosynthetic enzymes inhibition that result in indoleamine (serotonin) depletion was shown in the FS with p-chlorophenylalanine methyl ester (PCPA100 mg/kg, i.p.) pretreatment for four consecutive days followed by acute oral administration of vehicle, DF (250 mg/kg) or imipramine (30 mg/kg) as described under Section  2.4.2.Figure 4(a) showed a reduction in antidepressant-like effect of DF (^#^ *P* < 0.001) with PCPA pretreatment. Administration of this tryptophan hydroxylase (TrOH) inhibitor alone did not elicit significant behavioural alteration in the FS. Similar observation was made with the *α*-methyl-p-tyrosine (AMPT 100 mg/kg, i.p.) pretreatment, an inhibitor of tyrosine hydroxylase (TOH), the rate-limiting enzyme for catecholamine biosynthesis, administered 4 h before the FS.  Figure 4(b) shows blockade of DF anti-immobility effect by AMPT (_ _^#^ *P* < 0.05). Effect of DF on brain MAO as investigated showed, unlike clorgyline and tranylcypromine that reduced MAO activities to 17.7 ± 6.4% (*P* < 0.001) and 47 ± 9.0% (*P* < 0.01), respectively, its ineffectiveness to inhibit MAO activity (103.7 ± 8.8%, *P* > 0.05). With this result, it is obvious that DF do not have inhibitory effect on any of the MAO isoforms (A and B).

## 4. Discussion

Previous phytochemical analysis of the fractions obtained through liquid-liquid partitioning of the ethanol leaf extract of *P. pseudocaryophyllus* showed the presence of triterpenes, flavonoids, besides the (E)-methyl isoeugenol which constitute almost all of the essential oil (approximately 94%) [28]. In the dichloromethane fraction (DF), notable phytoconstituents identified with appropriate standards are oleanolic acid and methyl isoeugenol as earlier reported [8]. Being one of the major phytoconstituents found and isolated, quantitative analysis of oleanolic acid showed a relative composition of approximately 8%. Meanwhile, estimation of DF doses administered in present study is based on its effective dose in previously published [8] and unpublished data. 

The neuropharmacological activity of dichloromethane fraction (DF) had been demonstrated and was associated with the involvement of serotonergic pathway [8]. As earlier stated, investigation of antidepressant-like effects of DF is partly reinforced with the hypothesis that anxiolytic property of DF could be linked with its putative antidepressive action. In order to screen antidepressant effect of DF, tail suspension test (TS) was conducted. TS is a predictive and well-established animal model of antidepressant activity [21] that permits investigation of anti-immobility property of a novel molecule. Significant reduction in immobility time in TS and consistent reduction of this parameter in the forced swimming tests can be associated with antidepressant-like effect of this fraction. These results are similar to those obtained with imipramine treatment (norepinephrine/serotonin reuptake inhibitor) that is known to elicit an antidepressant response in FS [29]. FS remains one of the most effective and widely acceptable preclinical animal models [22, 30]. 

 Acute treatment with the standard drug or DF seems not to satisfy the aspect of face validity in this test considering the notion that therapeutic actions of antidepressant drugs evolve gradually with chronic treatment [31–33]. However, preliminary study showed that anti-immobility activity of DF at the doses tested in this work was not significantly different as compared to the chronic treatment (data not shown). This may be of clinical benefit as cases of nonadherence and risk attached to chronic treatment can be drastically reduced. In contrary to the position of some authors that immobility is an adaptive coping mechanism to conserve energy [34, 35], we share the opinion that immobility reflects behavioural despair and is a reliable means of demonstrating predictive validity [36, 37]. 

 The insinuations of acquirement of anxiolytic properties by antidepressants following chronic administration [38] may not truly represent the cellular processes involved. In this study, we were able to show in contrast to this assertion antidepressive effect of DF (a fraction that has shown anxiolytic-like property) with an acute treatment. In essence, this result can be attributed to the presence of active principles that are capable of eliciting antidepressive-like activity. Oleanolic acid among other constituents may have played a role in this activity as there are reports that demonstrated antidepressive effect of some triterpenes [39, 40] and linked oleanolic acid to CNS-mediated antinociceptive effect [41].

 However, the intriguing nature of DF 500 mg/kg (highest dose) insignificance effect in antidepressive models (TS and FS) led us to its evaluation in the open field. In this animal model, parameters (locomotor activity and rearing, which some authors considered as vertical movement) that are susceptible to the effects of myorelaxant or sedative agent were evaluated to augment information obtained on antidepressant models. Interestingly, reduction in these parameters as a result of DF 500 mg/kg administration is an indication of motor incoordination. Moreover, CNS stimulatory effect is also one of the commonly found false positive effects of natural or synthetic product in these models. However, previous results [8, 9] did not show any form of motor alteration (psychostimulatory or sedative) after DF 250 mg/kg oral treatment.

 In an attempt to investigate possible mechanism of action involved, biosynthetic enzymes was hypothesized to influence the synaptic level of monoamine. Metabolic activities of cytosolic enzymes like tryptophan hydroxylase (TrOH) and tyrosine hydroxylase (TOH) indirectly influence monoaminergic transmission. TrOH and TOH are rate limiting enzymes in serotonin and norepinephrine synthesis, respectively. Evidences in the literature showed that inhibition of norepinephrine and serotonin synthetic enzymes blocked antidepressant effect of desipramine or fluoxetine, respectively, and elicit a rapid return of symptoms in depressed patients [42, 43]. Serotonin and noradrenaline depletion approach has been utilized [44] in animal model to elicit depressive-like behaviour. Mice were depleted of serotonin with the parachlorophenylalanine (PCPA) (tryptophan hydroxylase inhibitor) for 4 days [45] while *α*-methyl-p-tyrosine (AMPT) was used to deplete catecholamine storage [43, 46]. Pretreatment with these biosynthetic enzymes inhibitors in this research abolished antidepressive-like response to DF treatment. These results are in agreement with the results in the literature that showed increase in affective disorder symptoms due to inhibition of monoamine synthesis by PCPA and AMPT [47, 48]. 

Reduction in immobility time in this research is a reflection of an increase in swimming and struggling. According to Millan [49], dual norepinephrine and serotonin reuptake inhibitors may produce persistent effects on both noradrenergic and serotonergic neurotransmission for greater efficacy and a more rapid onset of action. Anti-immobility response which could be regarded as a measure of physiological alterations to acute DF treatment may be associated with the development of synergy among neural pathways. 

Moreover, activity of drugs on MAO has been employed in the treatment of depression. In the treatment of this neural disease, monoamine oxidase inhibitors especially MAO A inhibitors (clorgyline, moclobemide) have proved to be more effective compared to MAO B inhibitor-like selegiline [50, 51]. MAO A is acknowledged for its preferential catabolic activity on 5-HT and NE (substrate). Research has also demonstrated antidepressant action of vast number of medicinal plant extracts among which is *Hypericum perforatum* that inhibits monoamine oxidase (A and B) [52]. Unlike DF-treated group, data obtained on MAO ex vivo assay showed significant reduction in enzymatic activity with the clorgyline and tranylcypromine treatment as compared to the vehicle treated group. Based on the experimental data and standard drugs used (clorgyline 15 mg/kg, an irreversible and selective inhibitor of monoamine oxidase A [53] and tranylcypromine 15 mg/kg, a nonselective and irreversible inhibitor of monoamine oxidase (MAO) [54]), we can infer that DF is not an effective inhibitor of MAO. 

In conclusion this work reveals antidepressive-like property of dichloromethane fraction and integrates new findings of possible mechanisms underlining antidepressant action with a growing body of evidence on vital role of monoamine biosynthetic enzyme. Subsequent preclinical study will be focused on active principles that are responsible, toxicological study, and dose extrapolation for possible clinical trial.
